# Minimally Invasive Surgery for Adolescent Idiopathic Scoliosis: A Systematic Review

**DOI:** 10.3390/jcm13072013

**Published:** 2024-03-29

**Authors:** Athanasios I. Tsirikos, Kaustubh Ahuja, Mohsin Khan

**Affiliations:** Scottish National Spine Deformity Centre, Royal Hospital for Children and Young People, Edinburgh EH16 4TJ, UK; kaustubh.ahuja@nhslothian.scot.nhs.uk (K.A.); mohsin.khan@nhslothian.scot.nhs.uk (M.K.)

**Keywords:** minimally invasive surgery, adolescent idiopathic scoliosis, thoracoscopic, vertebral body tethering

## Abstract

**Background:** Minimally invasive surgical (MIS) techniques have gained popularity as a safe and effective alternative to open surgery for degenerative, traumatic, and metastatic spinal pathologies. In adolescent idiopathic scoliosis, MIS techniques comprise anterior thoracoscopic surgery (ATS), posterior minimally invasive surgery (PMIS), and vertebral body tethering (VBT). In the current systematic review, the authors collected and analyzed data from the available literature on MIS techniques in AIS. **Methods:** The articles were shortlisted after a thorough electronic and manual database search through PubMed, EMBASE, and Google Scholar. **Results:** The authors included 43 studies for the review; 14 described the outcomes with ATS, 13 with PMIS, and 16 with VBT. **Conclusions:** While the efficacy of the ATS approach is well-established in terms of comparable coronal and sagittal correction to posterior spinal fusion, the current use of ATS for instrumented fusion has become less popular due to a steep learning curve, high pulmonary and vascular complication rates, implant failures, and increased non-union rates. PMIS is an effective alternative to the standard open posterior spinal fusion, with a steep learning curve and longer surgical time being potential disadvantages. The current evidence, albeit limited, suggests that VBT is an attractive procedure that merits consideration in terms of radiological correction and clinical outcomes, but it has a high complication and re-operation rate, while the most appropriate indications and long-term outcomes of this technique remain unclear.

## 1. Introduction

Posterior spinal instrumented fusion (PSIF) is the current gold standard for the surgical management of adolescent idiopathic scoliosis (AIS) [1]. The main objectives of surgery for AIS include prevention of deformity progression, correction of the scoliotic curve, restoration of global coronal and sagittal balance, as well as achieving fusion. While PSIF is safe and effective in accomplishing these objectives, it can be associated with excessive blood loss, long ICU and hospital stays, postoperative pain, and opioid requirements [2,3]. Paravertebral muscle injury resulting in denervation, fatty infiltration and atrophy are other consequences of the wide posterior exposure in the conventional PSIF procedure [4]. In addition, as with any major open surgery, PSIF carries a risk of surgical site infection [5].

Minimally invasive surgery (MIS) has gained popularity as a safe and effective alternative to open surgery for degenerative, traumatic, and metastatic spinal pathologies in the adult population [6,7,8]. Several researchers have employed minimally invasive techniques for the surgical management of idiopathic scoliosis in the form of anterior thoracoscopic (ATS) and posterior minimally invasive surgery (PMIS). ATS gained popularity in the 1990s up to the early 2000s and was eventually replaced by the posterior approach after the introduction of pedicle screw instrumentation [9]. Its current use includes definitive fusion for Lenke 1 and 5 curves, anterior release in severe scoliosis, and vertebral body tethering (VBT). VBT is a technique of growth modulation wherein a tether arrests the convex vertebral growth, allowing the remaining concave growth to achieve gradual scoliosis correction.

PMIS was first described in 2011 with the aim of reducing the invasiveness and morbidity associated with PSIF. However, several limitations restrict its widespread use. Common concerns associated with this procedure include technical difficulty in inserting pedicle screws and capturing the rods, excessive radiation exposure, and limited access to perform corrective maneuvers such as en bloc derotation, translation, and direct vertebral rotation [10,11]. The ability to correct the scoliosis is further impacted due to the limited approach provided by this technique to carry out facetectomies and wide spinal releases that can mobilize the spine before corrective maneuvers are employed. This can have an effect on achieving a fusion bed and subsequent arthrodesis, given the narrow inter-facetal exposure. 

The current study aims to provide a systematic review of the available literature encompassing the various options of using MIS techniques in the treatment of AIS focusing on their comparative outcomes. 

## 2. Materials and Methods

### 2.1. Literature Search

An extensive electronic and manual literature search of PubMed, EMBASE, and Google Scholar was performed using a combination of medical subject headings (MeSH) and text word databases. The search was aimed at identifying articles reporting safety and efficacy of minimally invasive surgery in scoliosis in the last 20 years. This review was not registered with any review registry. Medical subject heading (MeSH) terms used included “Scoliosis/surgery”, “Minimally Invasive Surgical Procedures”, and “Thoracic Surgery, Video-Assisted”, while non-MeSH search terms used included “Adolescent idiopathic scoliosis”, “early-onset scoliosis”, and “Vertebral body tethering”. Additionally, 2 authors cross-checked the references of the included citations to include any additional articles. The authors eliminated the duplicated citations first using Zotero’s (Fairfax, VA, USA) de-duplication function after merging all the articles followed by manual elimination.

### 2.2. Study Selection

The inclusion criteria for the articles were laid down following the PICOS format: (1) studies describing the outcomes of AIS patients undergoing surgical management (Population), with (2) minimally invasive approach (ATS, PMIS, or VBT) (Intervention), (3) with or without a comparison (open PSIF) group (Control), (4) reporting key components of intra-operative and postoperative clinical outcomes, radiological measurements, and complications of MIS in scoliosis (Outcomes), in (5) Study design; all studies except case reports or case series with a sample size of less than 5 were included. Studies with incomplete outcomes or data, meta-analysis, editorials, letters, and reporting outcomes in languages other than English were excluded. Any disagreements between the authors were resolved by means of discussion to reach a consensus.

### 2.3. Quality Assessment

The quality of the included studies was assessed using the NIH quality assessment tool by 2 authors. The tool assesses each study based on 9 pre-defined criteria including adequate description of research question, study population, outcome measures, statistical analysis and results, consecutiveness of cases, and comparability of subjects, with each criterion given 1 point if considered ‘Yes’ and 0 points if considered ‘no’. Each study was given a score out of 9 points. All studies scoring more than 6 were included in the analysis.

### 2.4. Data Collection Process and Synthesis Methods

Data extraction was carried out from the included citations by the first 2 authors. The study characteristics included the name of the first author, title, journal, year of publication, study design, level of evidence, and quality of the study. The extracted demographic information included the number of patients, mean age and gender ratio, and levels fused. The extracted operative and radiological data included the operation duration, blood loss, ICU/hospital stay, preoperative and final scoliosis angles, scoliosis angle correction, preoperative and final thoracic kyphosis (T5-T12), and complications. Once the data were extracted, the studies were classified under 3 headings: (1) anterior thoracoscopic fusion surgery (ATS), (2) posterior minimally invasive surgery (PMIS), and (3) vertebral body tethering surgery (VBT). Furthermore, attempts were made to contact the investigators in order to obtain detailed information if the data in the articles were incomplete or unclear.

## 3. Results

**Figure 1:**
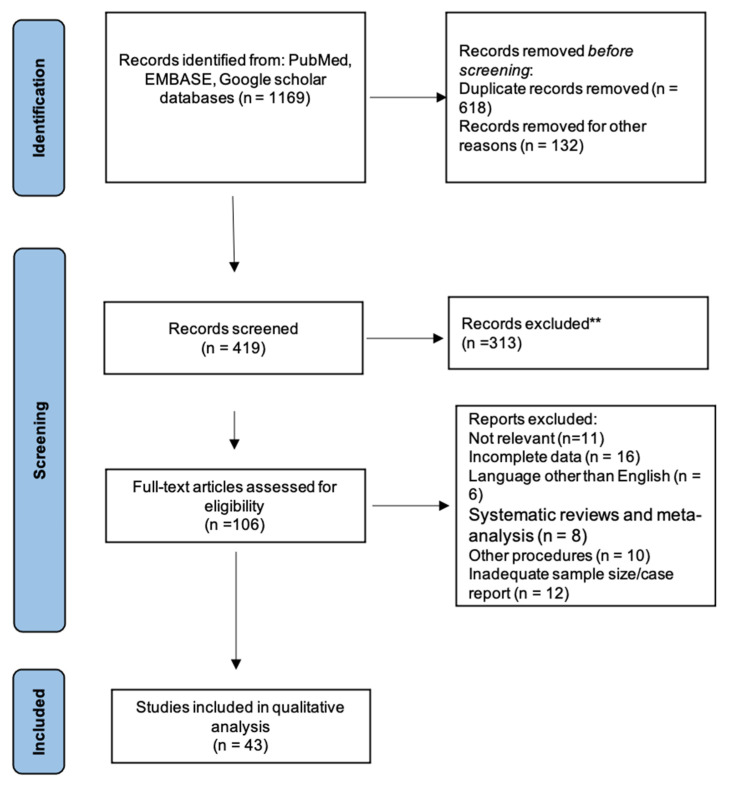
PRISMA flowchart showing study selection. ** Unable to fulfill the inclusion criteria.

A total of 1169 studies were identified across the various databases. After excluding duplicated and irrelevant articles, 419 articles were included for title and abstract screening, and 106 articles were subsequently reviewed as full texts to establish if they fulfilled the inclusion criteria. After assessing the complete texts, 39 articles were selected to be included in the study. Articles with inadequate sample size or data and publications in a language other than English were excluded. The PRISMA flowchart of study selection is shown in Figure 1. Out of these 43 studies, 14 described the outcomes of ATS, 13 of PMIS, and 16 of VBT.

### 3.1. Anterior Thoracoscopic Surgery

#### 3.1.1. Study and Curve Types

Fourteen studies were identified describing outcomes with ATS scoliosis surgery. Out of these fourteen studies, seven were case series [2,12,13,14,15,16,17], four studies compared ATS and thoracotomy approaches [18,19,20,21], two compared ATS with PSIF [22,23], and one compared all three approaches (ATS, thoracotomy, and PSIF) [24]. All but one study described the outcomes of treatment in patients with AIS. One study included an additional eight patients with neuromuscular scoliosis [13]. 

#### 3.1.2. Surgical Technique

All but two articles described the surgical techniques employed in their studies. All surgeries were performed with single lung ventilation in a lateral decubitus position with the convex scoliotic side facing up. Depending on the length of instrumentation, surgeon’s preference, and curve flexibility, 3–6 entry portals were used commonly at the third, fifth, seventh, and ninth ribs in order to insert T4-L1 instrumentation. If extension of the fusion was required to L3, an additional mini-retroperitoneal incision was necessary.

The parietal pleura was excised and the intervertebral segmental arteries were ligated or cauterized. Discectomies were performed before insertion of transvertebral screws. For bone grafting, autologous bone was harvested from the posterior superior iliac crest or ribs. Two studies used femoral head allografts to supplement the bone autograft [13,15]. Commonly used implant systems were the Eclipse or CD Horizon systems (Medtronic, Memphis, TN, USA), as well as the MOSS-Miami or Frontier systems (Depuy Synthes Spine, Raynham, MA, USA). Agarwal and Sucato [12] compared the awl-staple and guide-wire technique of screw insertion and concluded that the awl-staple technique was associated with shorter operative times, lesser blood loss, and fewer implant- and screw-related complications. Yoon et al. [25] compared a 4.75 mm titanium rod with a 4.0 mm stainless steel (SS) rod for anterior instrumentation in the context of high rates of pseudoarthrosis (between 5 and 31%) with the use of SS rods. The authors recorded fewer implant-related complications in the titanium group (21% vs. 8%) [25].

#### 3.1.3. Operative Outcomes (Table 1)

The studies comparing the anterior spinal approaches reported significantly shorter surgical times for the open procedure as compared to the thoracoscopic approach. In studies comparing anterior approaches and PSIF, posterior surgeries were found to be significantly faster than both open and thoracoscopic anterior procedures. 

**Table 1:** Table showing clinical outcomes for studies describing anterior thoracoscopic scoliosis (ATS) surgery.

Study	Year	Surgical Technique	Study Type	N	F	M	Age	Levels Fused	Surgical Time	Blood Loss	Transfusion Required	ICU Time	Hospital Stay
Sucato and Agarwal [12]	2008	Awl and Staple (ATS)	Case Series	15			13.4 ± 1.6	5.9 ± 0.6	416.1 ± 65.4	417.9 ± 268.6			
Sucato and Agarwal [12]	2008	Guide wire (ATS)	Case Series	27			13.7 ± 1.8	6.6 ± 0.9	505.6 ± 61.8	716.7 ± 371.3			
Gatehous [13]	2007	ATS	Case series	100	89	11	16.2	6.8	280	322			5.7
Grewal [18]	2005	OASF	Case Control	114			14 ± 3	7.7 ± 1.3	383 ± 65	924 ± 724		1.4 ± 1.2	10.4 ± 5.6
		ATS		41			14.3 ± 1.5	7.6 ± 0.7	508 ± 98	1218 ± 747 *		1 ± 0.3	11.3 ± 6.3
Kim [14]	2007	ATS	Case Series	42	34	8	15.6	5.9					
Lonner BS et al. [26]	2005	ATS	Case Series	57	42	15	15.1		342 ± 78	345.6 ± 165.9			4.7 ± 1.2
Norton et al. [15]	2007	ATS	Case Series	45				6.2	346	385			2.9
Picetti et al. [16]	2004	ATS	Case Series	50	40	10	12.7	7.8	366	267			2.9
Qiu et al. [19]	2008	ATS	Case Control	12	12	0	14.9	7.4 ± 1.3	390 ± 82	600 ± 155			
		Anterior mini thoracotomy		37	33	4	14.1	7.8 ± 0.9	170 ± 80 *	320 ± 120 *			
Newton et al. [17]	2005	ATS	Case series	50	44	6	14		350 ± 50	431 ± 273			6 ± 1
Newton et al. [24]	2013	ATS	Case- control	55				6	344 ± 103	470 ± 455 *	162 ± 266 *		6 ± 3
		OASF		17				7	400 ± 94	750 ± 472	210 ± 218		9 ± 4 *
		PSIF		64				10 *	238 ± 78 *	807 ± 608	365 ± 579		5 ± 1
Faro et al. [20]	2005	ATS	Case-control	31	30	1	13.3 ± 1.5	7.3 ± 0.8					
		Thoracotomy		23	19	4	14.8 ± 2.8	7.6 ± 0.8					
Lee et al. [22]	2013	ATS	Case Control	42	35	7	15.9 ± 3.8	6.3 ± 0.7	408 ± 90	876.2 ± 397.6 *	21.4% *		11.1 ± 2.4 *
		PSIF		23	20	3	14.8 ± 3.6	8.9 ± 0.9	282 ± 36 *	1632.6 ± 919.7	56.50%		8.7 ± 1.5
Wong et al. [23]	2004	ATS	Case control	12			14.3 ± 2.5	6.3 ± 0.7	415 ± 72	313 ± 363 *		2.6 ± 1.3 *	8.3 ± 1.2 *
		PSIF		19			14.4 ± 1.4	9.8 ± 1.5 *	252 ± 35 *	368 ± 285		1.5 ± 0.8	7.5 ± 0.9
Kishan et al. [21]	2007	ATS	Case-control	36			14 ± 2	6 ± 1					
		Thoracotomy		28			15 ± 3	6 ± 1					
		Thoracotomy with Thoracoplasty		43			14 ± 2	7 ± 1 *					

OASF: open anterior scoliosis fixation; PSIF: posterior spinal instrumented fixation; * denotes significance with *p* < 0.05.

With respect to blood loss, two studies reported significantly higher blood loss in the thoracoscopic approach as compared to the open anterior procedure [18,19], while one study noted no significant difference [24]. All three studies comparing outcomes in PSIF and anterior approaches reported significantly higher blood loss in the posterior as compared to the anterior approach [22,23,24].

The hospital stay in PSIF was significantly shorter than in the anterior surgeries. Among the open or thoracoscopic anterior approaches, the difference in hospital time was not significant. Five articles reported the steep learning curve with thoracoscopic procedures and improvement in operative outcomes with increasing experience [2,13,16,17,22]. All studies reported a negative correlation between the surgical time and the surgeon’s experience. Gatehouse et al. [13] reported significant improvements in operative time, set up time, radiation exposure, blood loss, and hospital stay in the last 20 AIS patients operated upon thoracoscopically when compared with the first and the middle 20 patients.

#### 3.1.4. Radiological Outcomes (Table 2)

All studies reported coronal curve correction. The reported scoliosis correction varied from 50 to 83%. The comparative studies revealed no significant difference in scoliosis correction with either approach. When comparing restoration of thoracic kyphosis, two studies reported significantly better outcomes in terms of preservation of thoracic kyphosis with the anterior approach when compared to PSIF [22,24], while one study reported no difference [23].

**Table 2:** Table showing radiological outcomes for studies describing anterior thoracoscopic scoliosis (ATS) surgery.

Study	Year	Surgical Technique	Pre-Op Scoliosis	2 Years Scoliosis	Flexibility	Correction	T5-T12 Kyphosis Pre-Op	Post-Op
Sucato and Agarwal [12]	2008	ATS (Awl and Staple)	54.3 ± 9.8		60.4 ± 17.1%	69.2 ± 14.5%		
Sucato and Agarwal [12]	2008	ATS (Guide wire)	57.2 ± 4.8		59.2 ± 14.3%	64.1 ± 11.3%		
Gatehouse [13]	2007	ATS	52.5	19.5		63%		
Grewal [18]	2005	OASF	48.5 ± 14°	17.5 ± 8°		64%		
		ATS	49.8 ± 7°	15.2 ± 7.5°		69%		
Kim [14]	2007	ATS	54.5 ± 13.9°	19.7 ± 9.3°		64%	18.2 ± 7.7°	22.4 ± 7.2°
Lonner et al. [26]	2005	ATS	48.3 ± 5.8°	19.3 ± 8.0°				
Norton et al. [15]	2007	ATS	51.6 (40–64)			83%		
Picetti et al. [16]	2004	ATS	58			50%		
Qiu et al. [19]	2008	ATS	52 (40–72)			65 ± 16%		
		Anterior mini thoracotomy	56 (42–72)			70 ± 12%		
Newton et al. [17]	2005	ATS	53 ± 9	24 ± 7	28 ± 10	52% ± 16	19 ± 10	28 ± 10
Newton et al. [24]	2013	ATS	48 ± 9		53 ± 19	57% ± 17	14 ± 10	+10 ± 10
		OASF	46 ± 6		48 ± 17	57% ± 18	16 ± 11	+10 ± 11
		PSIF	49 ± 7		52 ± 17	57% ± 15	21 ± 10 *	−4 ± 10 *
Faro et al. [20]	2005	ATS	54 ± 10	22 ± 6		59% ± 10	20 ± 9	26 ± 9
		Thoracotomy	57 ± 8	26 ± 9		54 ± 12	18 ± 16	27 ± 9
Lee et al. [22]	2013	ATS	52.2 ± 8.2	21.4 ± 9.8	63 ± 18	65.6 ± 13.2	21.7 ± 9.9	27.8 ± 9.2 *
		PSIF	55 ± 13	16.8 ± 8.7	54.9 ± 16.7	72.1 ± 11.8	20.1 ± 11.4	20.2 ± 9.3
Wong et al. [23]	2004	ATS	52 ± 11	20 ± 10	33 ± 12; 37% ± 15	62%	19 ± 12	26 ± 9
		PSIF	50 ± 9	16 ± 8	28 ± 11; 44% ± 14	67%	18 ± 13	23 ± 6
Kishan et al. [21]	2007	ATS	52 ± 9	21 ± 8		60 ± 13		
		Thoracotomy	55 ± 10	26 ± 12		52 ± 20		
		Thoracotomy with Thoracoplasty	52 ± 7	23 ± 10		58 ± 18		

OASF: open anterior scoliosis fixation; PSIF: posterior spinal instrumented fixation; * denotes significance with *p* < 0.05.

#### 3.1.5. Pulmonary Function Tests (PFTs) (Table 3)

Three comparative studies and one case series reported on PFTs [17,20,21,24]. One study compared the preoperative and 2-year postoperative PFT outcomes in three groups: anterior thoracoscopic fusion, open thoracotomy fusion, and open thoracotomy fusion with associated thoracoplasty [21]. This reported a decline of 1%, 8%, and 15% in forced vital capacity (FVC) and 2%, 5%, and 14% in forced expiratory volume in the first second (FEV1), respectively, in the three groups, with significant differences in favor of the anterior thoracoscopic when compared to the open thoracotomy with thoracoplasty group [21]. Among individual groups, the decline in lung function was significant in both the open thoracotomy and the open thoracotomy with thoracoplasty groups. Faro et al. [20] reported significantly greater decline in the open thoracotomy group in 3-month postoperative FVC. The values remained low in this group, while in the anterior thoracoscopic group, the values recovered. The decline in FEV1 values at 3 months post-surgery was similar in the 2 groups; however, by 1 year after surgery, the thoracoscopic group recovered more than the open thoracotomy group. Newton et al. [24] compared the posterior with anterior approaches and demonstrated a significant decline in the FVC in the open anterior compared to the posterior group.

**Table 3:** Table showing pulmonary function tests for studies describing anterior thoracoscopic scoliosis (ATS) surgery.

Study	Year	Surgical Technique	FVC	FEV1	FVC 2 YEARS	FEV1 2 YEARS
Newton et al. [17]	2005	ATS	2.9 ± 0.6 92% 18%	2.5 ± 0.5 84% 12%	3.1 ± 0.5 92% 15%	2.7 ± 0.4 84% 12%
Newton et al. [24]	2013	ATS	2.7 ± 0.5	2.3 ± 0.4	2.7 ± 0.5	2.4 ± 0.4
		OASF	2.8 ± 0.6	2.4 ± 0.5	2.5 ± 0.5	2.3 ± 0.5
		PSIF	3.1 ± 0.7	2.6 ± 0.6 *	3.3 ± 0.7	2.7 ± 0.6 *
Faro et al. [20]	2005	ATS	2.89 ± 0.49; 86% ± 15%	2,42 ± 0.45; 82% ± 14	2.82 ± 0.52; 85% ± 14	2.49 ± 0.44; 79% ± 12
		Thoracotomy	3.09 ± 0.64L	2.59 ± 0.53; 82% ± 14	2.83 ± 0.61 *	2.46 ± 0.52; 75% ± 11 *
Kishan et al. [21]	2007	ATS	2.9 ± 0.7; 87% ± 19	2.5 ± 0.6; 82% ± 16%	86% ± 18%	2.6 ± 0.6; 80% ± 16%
		Thoracotomy	2.9 ± 0.6; 83% ± 16	2.5 ± 0.5; 79% ± 17%	75% ± 17% *	2.5 ± 0.5; 74% ± 17% *
		Thoracotomy with Thoracoplasty	3.0 ± 0.8; 91% ± 17%	2.5 ± 0.6; 87% ± 15%	2.7 ± 0.7; 76% ± 11% *	2.5 ± 0.6; 73% ± 15% *

OASF: open anterior scoliosis fixation; PSIF: posterior spinal instrumented fixation; * denotes significance with *p* < 0.05.

#### 3.1.6. Patient Reported Outcome Measures (PROMs)

Two studies presented patient reported outcomes using the Scoliosis Research Society (SRS) questionnaire [2,24]. Newton et al. [24] reported significant improvement in PROMs in the postoperative period in all three groups (ATS, OASF, and PSIF). The comparison between the three groups did not reveal any significant difference. In contrast, Lonner et al. [2] reported significantly better SRS scores in the ATS group at final follow-up.

#### 3.1.7. Complications

All 14 studies reported complications associated with the procedure. The overall complication rates ranged from 8.3 to 54.5%. The most common were lung-related (9%) followed by instrumentation-related complications (7%) and non-union (2%). Among pulmonary complications, common pathologies were hemothorax, chylothorax, mucous plugs, atelectasis, and persistent pleural effusion, with 50% of complications requiring additional medical interventions. Implant-related complications included screw pull out, rod fracture, and slippage, with 33% of these complications requiring revision surgery. Four vascular complications were reported in three studies, including superior mesenteric artery syndrome in two patients (needing enteral feeds), aortic indentation in one patient, and segmental vessel bleeding needing conversion to a mini open thoracotomy in one patient [2,18,22].

### 3.2. Posterior Minimally Invasive Surgery

#### 3.2.1. Study and Curve Types

Thirteen studies were found which presented the outcomes of PMIS for scoliosis. Of these thirteen studies, nine were comparative studies [10,27,28,29,30,31,32,33,34], while four were case series [35,36,37,38]. All studies included only AIS patients.

#### 3.2.2. Surgical Technique

As reported in the included studies, the operation was performed in the prone position on a radiolucent table or an Allen frame. For thoracic curves, most authors have described performing 3 separate skin incisions whereas 1–2 incisions usually suffice for thoracolumbar/lumbar curves. A 2-inch-long incision usually allows instrumentation placement in three to four segments. The skin was undermined to obtain adequate exposure for a paramedian muscle-splitting approach which allowed pedicle screw insertion. The techniques described for pedicle screw insertion included free hand, fluoroscopy-assisted, or CT-guided, with the use of O-arm navigation (Stealth Station S8 Surgical Navigation System, Medtronic) [30]. Once the facet joint was exposed through the paraspinal sacrospinalis-splitting approach, most authors preferred to either perform a facetectomy or use a burr to find the entry point for screw placement. Decortication of these facets was then carried out to allow bone consolidation, as the facets form the majority of the fusion bed.

Passing of a contoured rod may be challenging through these incisions. Sarwahi et al. [10] described the use of alternate reduction screws with extended tabs on the screw tulip alternating with percutaneous screws with open reduction tubes. It is easier to pass the rod in the caudal-to-cephalad direction, as there is no conflict with the patient’s head during the procedure. Other authors have described using percutaneous screws with reduction tubes at every level [32,35,37]. The convex rod is introduced first and correction of the deformity is achieved using en bloc rotation, gradual segmental vertebra to rod reduction, as well as compression and distraction techniques. The small incision size and the use of MIS screw systems can make the correction techniques technically challenging [10,33]. After the convex rod is secured, the concave rod is captured using a similar reduction technique. A final intra-operative X-ray is taken to confirm the implant position before closure.

#### 3.2.3. Operative Outcomes (Table 4)

All but one study reported estimated blood loss for MIS procedures [38]. Among the comparative studies, all but one reported significantly higher blood loss with PSIF when compared to posterior MIS surgeries. However, in the above-mentioned study, the authors carried out a sub-analysis in 244 patients operated on by the same surgeon to eliminate surgeon-related differences and reported similar blood loss and transfusion rates in the two groups [33].

**Table 4:** Table showing clinical outcomes for studies describing posterior minimally invasive scoliosis (PMIS) surgery.

Study	Year	Surgical Technique	Study Type	N	F	M	Age	Levels Fused	Surgical Time	Blood Loss	Transfusion Required	ICU Time	Hospital Stay
Miyanji et al. [28]	2015	PMIS	Case-Control	23	20	3	16.8 ± 0.4	10.2	475.3 ± 13.2	261.5 ± 20.8			4.4 ± 0.15
		PSIF		23	19	4	16.4 ± 0.3	12.2	346.4 ± 15.64	471.7 ± 36.09			5.9 ± 0.20
Miyanji et al. [27]	2013	PMIS	Case-Control	16	14	2	16.8 ± 1.2		444 ± 89	277 ± 105 *			4.63 ± 0.96 *
		PSIF		16	15	1	16.4 ± 1.2		350 ± 76 *	388 ± 158			6.19 ± 1.68
Sarwahi et al. [10]	2016	PMIS	Case-Control	7	6	1	14.3	10 ± 1	8.98	600			8
		PSIF		15	13	2	15.2	13 ± 1	7.07 *	800			7
Si et al. [29]	2021	PMIS	Case-Control	64	44	20	12.4 ± 1	8.4 ± 2.3	361 ± 95	502 ± 218 *	26.60% *		
		PSIF		48	34	14	14.7 ± 2.4	6.2 ± 2.6	275 ± 43 *	808 ± 520	58.30%		
Urbanski et al. [30]	2019	PMIS	Case-Control	4			15.5 ± 2.06	6.5 ± 0.86	285 ± 47.56	138.75 ± 50 *			3.75 ± 0.43 *
		PSIF		4			21.25 ± 9.98	5.75 ± 0.43	242.5 ± 44.51	450 ± 106			7 ± 3
Zhu et al. [31]	2017	PMIS	Case Control	15	13	2	16.5 ± 1.6	4.9 ± 0.5	252 ± 96	153 ± 97 *			
		PSIF		30	27	3	15.1 ± 1.7	5.7 ± 0.5	192 ± 30 *	418 ± 126			
Yang et al. [38]	2021	PMIS	Case Control	24			15 ± 1.9	12.3 ± 1.4	441 ± 107	1279 ± 725 *		12 ± 1.9	12.0 ± 1.9 *
		PSIF		25			14 ± 1.5	12.1 ± 1.5	287 ± 75 *	2503 ± 135		16.2 ± 3.7	16.2 ± 3.7
Sarwahi et al. [33]	2021	PMIS	Case Control	192	87%		15	11	279 (222.25–339.75)	350	5 (2.6%)		4 *
		PSIF		293	77.4%		15	12	259 (224–321) *	300 *	40(13.7%)		5
Syundyukov et al. [34]	2023	PMIS	Case Control	35	31	4	16.1 ± 2.2	10.4 ± 0.8	346.2 ± 70.5	208.7 ± 113.4 *			8.1 ± 1.6 *
		PSIF		47	44	3	15.7 ± 1.5	10.7 ± 1.7	266.6 ± 64.3 *	564.3 ± 242.7			11.2 ± 1.4
de Bodman et al. [36]	2020	PMIS	Case Series	93			15.2 ± 2.2		317 ± 106	322 ± 170			
Bodman et al. [35]	2017	PMIS	Case series	70			15 ± 10.1		337.1 ± 121.3	345.7 ± 175.1			
Yang et al. [37]	2020	PMIS	Case Series	84	77	7	15.2	10.7	312.8	846.6		0	8.5
Yang et al. [38]	2021	PMIS	Case Series	34									

PMIS: posterior minimally invasive scoliosis surgery; PSIF: posterior spinal instrumented fixation; * denotes significance with *p* < 0.05.

With respect to operative time, all but one comparative study reported significantly greater surgical time for MIS surgery. The only study reporting similar operative duration was that by Urbanski et al. [29] and included only Lenke 5C curves. To evaluate the learning curve for the procedure, Yang et al. [37] reported the outcomes by chronologically dividing the patients into two groups. The authors reported significantly lower surgery times and blood loss in patients operated on in the latter group within their study [38].

Hospital stay was reported in seven out of nine studies. Six of these seven studies reported significantly shorter hospital stays for patients undergoing posterior MIS surgeries. The only study reporting no significant difference was one of the earlier studies with a small sample size [10].

#### 3.2.4. Radiological Outcomes (Table 5)

All the included studies reported significant curve correction after surgery that was maintained at last follow-up as compared to the preoperative scoliosis angle. On analyzing comparative studies, seven articles [10,27,28,29,30,31,33] reported comparable scoliosis correction between the two groups, whereas two studies [34,38] reported significantly better correction achieved with PSIF. All studies reported changes in thoracic kyphosis measurements with contrasting results. Six out of nine studies reported no difference in kyphosis restoration, one study reported significantly better kyphosis correction with PMIS [33], while two studies reported better results with PSIF [34,38].

**Table 5:** Table showing radiological outcomes for studies describing posterior minimally invasive scoliosis (PMIS) surgery.

Study	Year	Surgical Technique	Study Type	Pre-Op Scoliosis	2 Years Scoliosis	Flexibility	Correction	T5-T12 Kyphosis Pre-Op	Post-Op
Miyanji et al. [28]	2015	PMIS	Case-Control	56.7 ± 1.6	23.9 ± 1.6		58.1 ± 2.4%	20.5 ± 2.0	22.9 ± 1.9
		PSIF		58.1 ± 1.5	18.7 ± 1.0		68 ± 1.4% *	22.6 ± 3.3	21.0 ± 1.3
Miyanji et al. [27]	2013	PMIS	Case-Control	56 ± 5	20 ± 8		63% ± 13%		21 ± 9
		PSIF		56 ± 8	18 ± 4		68% ± 8%		17 ± 5
Sarwahi et al. [10]	2016	PMIS	Case-Control	48			79.20%	22	24
		PSIF		46			84.70%	24	21
Si et al. [29]	2021	PMIS	Case-Control	50.7 ± 8.8	17.4 ± 8.5	50.2 ± 13.1	65 ± 17.6%	29.2 ± 9.4	28.7 ± 7.1
		PSIF		48.0 ± 8.4	17.2 ± 10.4	50.9 ± 8.9	64.4% ± 19.7	17.5 ± 8.8	17.8 ± 8.2
Urbanski et al. [30]	2019	PMIS	Case-Control	57.25 ± 10.64			68.25 ± 6.18	23.6 ± 7.61	26.075 ± 8.53
		PSIF		47 ± 7.78			78.25 ± 8.84	37 ± 16.06	32.4 ± 12.51
Zhu et al. [31]	2017	PMIS	Case Control	48.3 ± 4.2	11.1 ± 4.3	71.2 ± 8.75%	77.1 ± 8.9%	20.2 ± 6.1	25.2 ± 6.2
		vs. PSIF		50.9 ± 5.4	12 ± 3.1	73.3 ± 15.6%	76.5 ± 7.0%	16.5 ± 6.8	22.9 ± 7.5
Yang et al. [38]	2021	PMIS	Case Control	60.8 ± 9.4	22.5 ± 5.8	31.7 ± 17.7		35.0 ± 9.2	35.2 ± 8.5
		PSIF		62.1 ± 12.9	17.7 ± 5.0 *	29.9 ± 16.8		26.5 ± 12.1	29.3 ± 7.1 *
Sarwahi et al. [33]	2021	PMIS	Case Control	55	17		69.10%	25°	31° (23–35)
		PSIF		53	17		67.70%	25°	23° (17–29.6) *
Syundyukov et al. [34]	2023	PMIS	Cse Control	52.2 ± 11.3		22.45 ± 10.74	77.7 ± 10.7	17.7 ± 10.0	14.3 ± 10.2 *
		PSIF		53.4 ± 11.7		29.17 ± 14.06	88.2 ± 8.0 *	13.8 ± 6.2	18.4 ± 4.8
de Bodman et al. [36]	2020	PMIS	Case Series	58.4 ± 12.1	20 ± 7.5			26.5 ± 12.7	30.6 ± 8.9
Bodman et al. [35]	2017	PMIS	Case series	58.9 ± 12.6			69.4 ± 20.2	24.2 ± 12.2	30.1 ± 9.6
Yang et al. [37]	2020	PMIS	Case Series	59.8 ± 6.5	18.6 ± 4.71			31.2 ± 8.01	35.3 ± 6.35
Yang et al. [38]	2021	PMIS	Case Series	61.3°		26.10%	65.2%		

PMIS—posterior minimally invasive scoliosis surgery; PSIF—posterior spinal instrumented fixation; * denotes significance with *p* < 0.05.

#### 3.2.5. Patient Reported Outcome Measures

Three out of thirteen studies reported the SRS-22 scores [29,31,38]. There was no significant difference in activity, self-image, satisfaction, and mental health domains between the two groups. However, two of three studies showed significantly better pain scores with MIS [29,31].

#### 3.2.6. Complications

Of the included studies, six comparative studies and two case series reported complications. In the current study, total complication rate was found to be 21.2% and 18.9% in PMIS and PSIF groups, respectively. None of the studies reported any difference in the prevalence of complications among the PMIS and PSIF groups. Commonly reported complications included surgical site infection, instrumentation failure, wound dehiscence, pseudarthrosis, and hemothorax. Surgical site infection was the most common complication (4.6% in PMIS vs. 5.4% in PSIF). The authors placed special emphasis on complications related to implant failure, as this may indicate a non-union. None of the studies reported any difference in the rate of instrumentation failure between the open and MIS posterior correction techniques.

### 3.3. Vertebral Body Tethering (VBT)

#### 3.3.1. Study and Curve Types

The authors identified 16 studies describing VBT. Of the sixteen VBT studies, twelve were case series [39,40,41,42,43,44,45,46,47,48,49,50] and four were comparative studies, with two studies comparing the outcomes of VBT with PSIF [51,52], one comparing VBT with PSIF and magnetic growing rods [53], and one comparing VBT with anterior spinal fusion [54].

#### 3.3.2. Surgical Technique

The position of the portals and endoscopic approach is similar to the technique described in the ATS. For insertion of the spinal instrumentation, a 5 cm muscle sparing thoracic incision was made at the eighth intercostal space. Three portals were created along the anterior axillary line between the fourth and eighth intercostal spaces, and one 10 mm portal was made medial to the mini-thoracotomy for placement of the thoracoscope [49]. In VBT, the procedure is very similar to ATS, except the rod was replaced by a polyethylene terephthalate (PET) tether (Zimmer, Biomet, Warsaw, IN, USA) placed in the proximal-to-distal direction and sequentially tensioned with a dynamometric set until 300 Nm. A chest tube was placed for the first 2–3 postoperative days.

Apart from Zimmer, other manufacturers are also developing VBT systems. The REFLECT scoliosis correction system developed by GLOBUS (Audubon, PA, USA) received FDA approval in 2023, although no literature has been published describing the use of this system. Similarly, Medtronic’s BRAIVE system’s (Dublin, Ireland) IDE study was also initiated in 2021, and its preliminary outcomes are awaited.

#### 3.3.3. Surgical Outcomes

In the included studies, the mean number of instrumented vertebral levels was 7.6. The mean operative time and blood loss was described in nine out of sixteen studies. The cumulative mean surgical time was 223 min and the mean blood loss was 144 mL. The hospital stay was reported in seven studies and the cumulative mean was 4.9 days.

#### 3.3.4. Radiological Outcomes (Table 6)

All studies reported a significant reduction in the mean scoliosis angle. A recent single-arm meta-analysis has reported a mean preoperative scoliosis angle of 47.8°, correcting to 22.2° at final follow-up. The comparative studies reported significantly better coronal curve correction with PSIF compared to VBT. Two articles reported on restoration of thoracic kyphosis, with contradictory results, as Newton et al. [51] favored PSIF over VBT and Mackey et al. [52] suggested better sagittal thoracic correction with the VBT technique [53].

**Table 6:** Table showing clinical and radiological outcomes for studies describing vertebral body tethering (VBT) surgery.

Author	Year	Study Type	*n*	Females	Age	Levels Tethered	Follow-Up Duration	Pre-Op Scoliosis	Final Scoliosis	Pre-Op Kyphosis	Final Kyphosis
Hoernschemeyer et al. [39]	2020	Case Series	31		12.7	7.2		50	9		
Pehlivanoglu et al. [40]	2020	Comparative	21	15	11.1	7.1	27.4	48.2	10	26.8	26
Newton et al. [52]	2020	VBT	23	16	12		24–60	53	33	25	19
		PSIF	26	23	13		24–60	54	16 *	25	29 *
Alanay et al. [41]	2020	Case Series	31	29	12.1	7.5	12–24	47	17		
Samdani et al. [42]	2021	Case Series	57	49	12.4	7.5	55.2	40.4	18.7	15.5	19.6
Rushton et al. [43]	2021	Case Series	112	104	12.7	7.3	37	50.8	25.7	18.6	21.4
Abdullah et al. [44]	2021	Case Series	120	107	12.6		24	51.2	27.5		
Baker et al. [45]	2021	Case Series	17	12	12.9		24	45	20	20	17
Baroncini et al. [46]	2021	Case Series	86	72	13.2	8.5	24	52.4	28.5	28	33
Buyuk et al. [47]	2021	Case Series	32	30	13	8	12	51	26	16	19
Yucekul et al. [48]	2021	Case Series	28	23	12.2	7.7	38.6	46	12		
Bernard et al. [54]	2022	Case-Control	10	10	12.5	7	64.5	47.4	19.4		
Costanzo et al. [49]	2022	Case Series	23	19	9–14 y	7	10–30 m	56.5	37		
Mackey et al. [53]	2022	VBT	37	36	11.3		36	50	28	26.1 *	25
		MCGR	51	35	9.6 *		34.8	64.5 *	42 *	34.7	34.2 *
		PSIF	42	34	10.9		43.2	63	29	35.9	25.8
Miyanji et al. [50]	2020	Case Series	57	54	12.7	7.3	40.4	51		18.7	22
Newton et al. [51]	2022	VBT	237	199	12.1		24.4	48	27	18	20
		PSIF	237	198	13.4 *			53 *	20 *	19	21

VBT: vertebral body tethering; MCGR: magnetically controlled growing rod; PSIF: posterior spinal instrumented fixation; * denotes significance with *p* < 0.05.

#### 3.3.5. Patient Reported Outcome Measures

Altogether, two studies have reported SRS-22 scores in patients undergoing VBT [40,52]. While one study reported comparable PROMs between patients undergoing VBT and PSIF [52], the other reported significantly better HRQoL and satisfaction scores in the VBT group [40].

#### 3.3.6. Complications (Table 7)

Sixteen studies reported complications, with a mean overall incidence of 23%. Tether breakage (22.3%), scoliosis over-correction (4.2%), and pulmonary complications (7.1%) were among the most commonly reported complications associated with VBT. Of the total complications, 13% required unplanned revision surgeries for their management.

**Table 7:** Table showing complications in studies describing vertebral body tethering (VBT) surgery.

Author.	Year	Study Type	Over-Correction	Tether Breakage	Pulmonary	Revision	Total
Hoernschemeyer et al. [39]	2020	Case Series	2	14	1	6	23
Pehlivanoglu et al. [40]	2020	Comparative	0	1	1	1	2
Newton et al. [52]	2020	VBT	3	12	3	9	18 *
		PSIF	0	0	0	0	0
Alanay et al. [41]	2020	Case Series	6	1	4	2	13
Samdani et al. [42]	2021	Case Series	5	NR	0	7	7
Rushton et al. [43]	2021	Case Series	5	36	9	18	28
Abdullah et al. [44]	2021	Case Series	2	4	4	7	19
Baker et al. [45]	2021	Case Series	0	9	NR	4	9
Baroncini et al. [46]	2021	Case Series	0	5	5	5	10
Buyuk et al. [47]	2021	Case Series	1	5	2	1	8
Yucekul et al. [48]	2021	Case Series	6	5	2	4	19
Bernard et al. [54]	2022	Case-Control	3	1	0	1	5
Costanzo et al. [49]	2022	Case Series		2	1	1	3
Mackey et al. [53]	2022	VBT		5		10	10
		MCGR	NA	10		33	33 *
		PSIF	NA	1		4	6
Miyanji et al. [50]	2022	Case Series	1	24	4	8	16
Newton et al. [51]	2022	VBT	16	47	NR	46	63
		PSIF	NA	NA	0	4	4

VBT: vertebral body tethering; MCGR: magnetically controlled growing rod; PSIF: posterior spinal instrumented fixation; * denotes significance with *p* < 0.05.

## 4. Discussion

The current systematic review was designed to analyze the available evidence on the use of MIS techniques and their outcomes for AIS. There is currently sufficient literature available to establish the safety and efficacy of anterior thoracoscopic and posterior MIS techniques for scoliosis.

### 4.1. Anterior Thoracoscopic Surgery

Anterior scoliosis correction was fairly prevalent before the advent of pedicle screw instrumentation. The current review investigated the reported outcomes of the anterior thoracoscopic approach in comparison to the PSIF and open anterior thoracotomy. The primary outcome measure for the majority of studies was scoliosis correction, and anterior thoracoscopic surgery showed results comparable to PSIF or open anterior thoracotomy and fusion. With respect to the sagittal spinal contour, restoration of thoracic kyphosis was reported to be better with ATS compared to PSIF. Lee et al. [22] reported significant improvement in the T5-T12 kyphosis (mean 21.7° corrected to mean 27.8°) with the anterior thoracoscopic approach as compared to PSIF (mean 20.1° corrected to mean 20.2°). Newton et al. [24] in his three-pronged comparative study reported significantly higher loss of kyphosis with posterior surgery as compared to the anterior corrective procedures. As with previous reports, these studies reiterated the kyphogenic effect of anterior surgery due to shortening the anterior vertebral column [14,18].

The clinical outcomes of ATS studies were comparable if not better than PSIF. Lonner et al. [2] reported higher SRS-22 outcomes in the domains of self-image, mental health, and total scores among patients undergoing ATS compared to PSIF. The activity, pain, and satisfaction domains, however, did not show any significant difference between the 2 groups. Padhye et al. [54] in their systematic review reported an average of 2.7 lesser spinal levels fused with anterior thoracoscopic correction when compared to PSIF. As saving vertebral levels with anterior scoliosis correction refers primarily to the most caudal end of the instrumentation, this can reduce the risk of distal degeneration while maintaining higher spinal mobility and improved patient functionality.

The advantages of ATS should be weighed against its potential complications, which include lung-related problems, implant failure, and non-union, as these can significantly increase patient morbidity. Padhye et al. [55] reported 44 pulmonary complications in 488 patients (9%) including hemothorax, chylothorax, mucous plugs, atelectasis, and persistent pleural effusion. Of these complications, at least 22 were reported to need additional procedures, including bronchoscopy and chest tube insertion, for resolution. In the same cohort of patients, the rate of instrumentation-related complications was 7% (34 of 488 patients), including rod breakage, slippage, and screw pull-out; 15 of these patients were reported to need a re-operation [54]. Finally, the steep learning curve and need for adequate theater set-up to support such a technique should be carefully assessed before choosing anterior thoracoscopic surgery as the preferred fusion technique. It is of note that several authors have reported reduced intra-operative blood loss, surgical time, and rate of complications with increasing surgeon experience [2,13,17,22]. Two important technical limitations should be kept in mind when considering the ATS technique. Firstly, the majority of ATS evidence is limited to the treatment of single thoracic AIS. Secondly, the upper extent of instrumentation is limited by the presence of the major vessels to the T4 vertebra [55].

### 4.2. Posterior MIS

MIS for AIS was conceptualized after the success of MIS in adult patients with degenerative, traumatic, and metastatic spinal conditions. Sarwahi et al. [55] first described the technique of posterior MIS in 2011 for scoliosis through three small midline skin incisions and utilizing the laxity of the skin to instrument up to three vertebral segments and employ common reduction maneuvers effectively. Since then, several authors have published their outcomes with MIS for scoliosis. Miyanji et al. [26], in a comparative analysis, reported significantly lower blood loss and shorter hospital stay with similar deformity correction achieved but prolonged surgery time when compared with conventional PSIF. Sarwahi et al. [55], in a large multi-center comparative study, reported similarly positive outcomes. Moreover, PMIS has been able to achieve a better kyphogenic effect with improved restoration of thoracic sagittal balance than open PSIF [33]. Their probable explanation was preservation of posterior spinal musculature. In one of the studies, patients in the MIS group were found to score better that the open PSIF in self-image and pain domains in the SRS-22 patient reported outcomes [29,31]. This could be explained by the smaller-sized incisions and intact spinal musculature.

Despite the advantages associated with MIS, such as less blood loss, shorter hospital stays, and similar correction to PSIF, several important concerns must be acknowledged. Longer operative time was a consistent finding in all the included studies. De Bodman et al. [35] and Zhu et al. [30] reported significant improvement in surgical time with increasing surgeon experience. One of the important concerns with MIS is high radiation exposure for the surgeon and the patients. Zhu et al. [30], in their study, used O-arm navigation to employ PMIS in the treatment of thoracolumbar/lumbar scoliosis and found no significant difference in curve correction when compared with PSIF. The other concern is the ability to achieve fusion with the limited exposure of the facet joints, inter-laminar and inter-transverse space that the PMIS technique allows, as this limits the area available for bone grafting. While none of the current studies reported any difference in instrumentation-related complications between the MIS and open posterior fusion techniques, longer follow-up studies are needed to establish the effectiveness of PMIS in maintaining scoliosis correction and achieving fusion at long-term follow-up. Although several reviews have analyzed the safety and efficacy of PMIS, the authors noticed considerable inconsistencies with respect to the number of studies, inclusion criteria of the studies, and their reported results [56,57,58]. The authors have tried to include all the available literature and report an unbiased systematic review of the results in the current study.

### 4.3. Thoracoscopic Anterior Vertebral Body Tethering

There has been a recent resurgence of the endoscopic technique in the management of AIS in the form of VBT. This non-fusion technique is based on the Heuter–Volkmann principle, where a mechanically tensioned convex polyethylene tether is expected to arrest the physeal growth on the deforming side of the curve while preserving concurrent growth on the contralateral concave side, allowing gradual spontaneous curve correction with further spinal development. The current FDA indications for this procedure include a skeletally immature patient (Sanders bone age ≤ 5 or Risser grade ≤ 2) of age 8–16 years with a major scoliosis angle of 35–60° involving thoracic, lumbar, or thoracolumbar curves that failed or did not tolerate bracing [59,60]. In its early stages, VBT was performed through an open thoracotomy or a mini-open approach [61]. However, most of the recent case series have described a thoracoscopic approach for this procedure [62]. Of the articles shortlisted for this review, only three performed a comparative analysis of VBT with PSIF. In one of these three, Pehlivanoglu et al. [39] reported a better range of motion, bending flexibility, extensor trunk endurance, and muscle strength, with significantly higher average total SRS-22 and 36-Item Short Form Survey (SF-36) mental component score (MCS)/Physical component score (PCS) scores when compared with age-, gender-, fusion level-, and minimum follow-up-matched PSIF cohorts. Interestingly, this study did not report any complication in either group. The other comparative study by Newton et al. [51] reported significantly less initial scoliosis correction, worse thoracic deformity at last follow-up (27 ± 12° vs. 20 ± 7°), more revision surgeries (16% vs. 1.3%), and less improvement in the pain and self-image scores in the VBT group as compared to the PSIF group. However, in this study, the VBT group included younger patients with smaller mean thoracic scoliosis. Due to lack of good-quality comparative studies, the VBT technique was discussed as a separate section in the current review.

Several authors have reported the efficacy of the VBT procedure. Samdani et al. [41] reported a mean scoliosis correction of 70% for main thoracic and 71% for lumbar curves at final follow-up. Newton et al. [51] reported a correction of 51% for thoracic curves in their study, with a mean follow-up of 2.5 years. In another series of 108 thoracic VBT procedures performed thoracoscopically with a mean follow-up of over three years, the scoliosis correction rate reported at the end of one year was 55.1 ± 22.7%, with a decline to 49.6 ± 30.5% at final follow-up. The authors attributed this loss of scoliosis correction over time to tether breakage [43].

While several case series have reported good radiological efficacy for VBT, a high rate of complications and re-operations is one of the main concerns for the technique. A recent meta-analysis reported tether breakage, scoliosis over-correction, and pulmonary complications as the most common problems associated with the procedure [63]. The pooled complication and re-operation rates reported in studies with a follow-up of less than 36 months were 11.8% and 2.9%, which increased to 25.2% and 24.7%, respectively, during longer follow-up, suggesting a considerable increase in the complication rates over time [40,43,51,52]. Most common indications for revision surgery included curve over-correction and tether breakage. Finally, the VBT to PSIF conversion rate was reported to be 1.4% due to deformity progression despite tethering. Other reviews have also reported similar complication and re-operation rates [64,65].

### 4.4. Posterior Vertebral Body Tethering

A recent novel modification of the VBT is the posterior vertebral pedicular tethering technique. The procedure has been described by Jorge Mineiro [66] for thoracolumbar/lumbar AIS with scoliosis angle between 40 and 60° and Sanders maturity grades 3–5. The biomechanical principles of this technique are similar to the anterior VBT using the Heuter–Volkmann law with growth plate compression inhibiting convex spinal growth, allowing remaining concave growth to correct the deformity over time. It involves placement of segmental pedicle screws using a paraspinal approach with a midline skin incision followed by insertion of a polyethylene tether. Tightening of the tether is performed using a tensioning device. The author presented his preliminary experience over a small series of six patients reporting scoliosis correction from a preoperative angle of 51.6° to 16.7° at latest follow-up. The only reported complication was over-correction in one patient, which required division of the tether. The author acknowledges the need to validate these results with longer follow-up and larger patient sample size.

The attraction of this technique is that it is performed through a posterior approach to the spine which most surgeons are familiar with, thus preventing the risk of respiratory and vascular complications. In addition, any re-operation is much easier to perform and avoids the additional risks of the anterior VBT, which include working in the anterior spinal column in an area of increased scarring from the index surgery that significantly increases the risk of severe injury to the lungs and major vessels. It is likely that we will hear more on the posterior vertebral body tethering technique in the future and as the indications and limitations of this new technique are better clarified.

## 5. Limitations

The current study has a few limitations. The authors did not perform a meta-analysis of the acquired data. During data analysis, significant heterogeneity was observed in terms of study designs, curve types, correction techniques, management protocols at different centers, and most importantly, reporting of outcomes. This heterogeneity can have a significant impact on generating a meaningful analysis. In addition, in the VBT review, the majority of studies included were case series, thus lacking a control group. While it is possible to establish the safety and efficacy of this technique, a comparison with the conventional fusion technique is not possible. Finally, with respect to PMIS, while several studies have emphasized the importance of a learning curve and improvement of outcomes after the first 25 cases [36], in our data, six of nine comparative studies have reported their outcomes in their first 25 or fewer cases.

## 6. Conclusions

In conclusion, while the efficacy of ATS is well-established in the treatment of AIS in terms of comparable coronal and perhaps better sagittal deformity correction than PSIF, the current use of anterior thoracoscopy for fusion has become less popular due to a steep learning curve, considerable pulmonary and vascular complications, implant failures, and high non-union rates. With respect to PMIS, although it is a safe and effective alternative to standard open PSIS, the steep learning curve, longer operative time, and appropriate patient selection are important considerations for this procedure. Multi-center large cohort studies with longer postoperative follow-up are required to better understand the rate of complications and balance these over the benefits of the procedure.

In regard to VBT, the current evidence suggests that VBT is a valid procedure in terms of radiological scoliosis correction and clinical outcomes in AIS patients. The added advantage of maintaining some spinal flexibility and muscle strength over PSIF, which produces a rigid spine, is particularly attractive for young patients. However, the high rate of complications and re-operations suggests the importance of optimizing the technique. There is a need to define the appropriate indications of the VBT technique and choose the optimal surgical candidates in regard to curve type, location and size of scoliosis, as well as remaining spinal growth that can achieve more reproducible outcomes and reduce the need for revision surgery. The risks of re-operation in the chest cavity or in the abdominal retroperitoneal space to gain access to the anterior vertebral column in the presence of extensive adhesions following the primary tethering procedure cannot be underestimated and the presence of an access surgeon is mandatory. The current literature lacks good-quality trials and comparative studies of VBT with other techniques of growth modulation and PSIF to allow a comprehensive analysis of its safety and efficacy.

## Data Availability

Any further data may be produced upon request.
